# Covid-19 and its impact on immunization programs: reflections from Brazil

**DOI:** 10.11606/s1518-8787.2020054003042

**Published:** 2020-11-04

**Authors:** Camila Carvalho de Souza Amorim Matos, Carolina Luísa Alves Barbieri, Marcia Thereza Couto

**Affiliations:** I Universidade de São Paulo Faculdade de Medicina Departamento de Medicina Preventiva São PauloSP Brasil Universidade de São Paulo. Faculdade de Medicina. Departamento de Medicina Preventiva. São Paulo, SP, Brasil; II Universidade Federal de Santa Catarina Departamento de Ciências da Saúde AraranguáSC Brasil Universidade Federal de Santa Catarina. Departamento de Ciências da Saúde. Araranguá, SC, Brasil; III Universidade Católica de Santos SantosSP Brasil Universidade Católica de Santos. Santos, SP, Brasil

**Keywords:** Coronavirus Infections, Immunization, Immunization Programs, Unified Health System, Health Care Systems

## Abstract

Due to social distancing guidelines and the displacement of both human and material resources to fight the covid-19 pandemic, individuals seeking healthcare services face certain challenges. Immunization programs have already been a worrisome topic for health authorities due to declines in vaccine uptake rates and are now especially affected by the covid-19 pandemic. Disbelief in science, dissemination of fake news about vaccines, socioeconomic vulnerability and social inequality are some of the challenges faced. This commentary article discusses the impacts of the covid-19 pandemic on immunization programs in Brazil. In light of advances (and notability) of Brazil's national immunization program, established in the 1970s, the programs face challenges, such as the recent drop in vaccine uptake rates. In addition to this health crisis, there is also Brazil's current political crisis, which will undoubtedly require assistance from researchers, policymakers and society to be fixed.

## INTRODUCTION

Covid-19, a disease caused by the novel coronavirus named SARS-CoV-2, has spurred discussions on how health systems are organized around the globe^1^. The current epidemiological scenario has revealed unexpected weaknesses in the health care response to this crisis, as in the case of the English National Health Service (NHS)^2^. In contrast, some countries with fragile health systems have shown rapid responses to the epidemic, as a result of lessons learned from previous health crises^3^.

Many factors have contributed to the success (or lack thereof) of each country in facing the pandemic so far. Some of those factors include the seriousness with which the government has faced the situation; the intensity and timing of social distancing; the assistance capacity in tertiary care level (especially ICU); the testing capacity to a greater or lesser extent, among others^4^. Countries with universal health systems tend to show better responses to the crisis^1^^,^^5^.

Humanitarian and economic repercussions of the pandemic are expected to weaken immunization programs around the world^6^. Governmental and international health agency officials worry about these immunization programs due to the growing phenomenon of vaccine hesitancy^7^.

As researchers reflecting upon Brazil's healthcare system and immunization programs, we asked ‘What can we learn from other countries in this current scenario?’

The interdependence of vaccine production and distribution processes among countries is a matter of global concern. An intercontinental vaccine ‘traffic’ crisis caused by an external factor of high magnitude, such as the covid-19 pandemic, can lead to a shortage of vaccines^8^. Can the large-scale production of a future vaccine, promoted by the WHO as necessary for global public welfare, lead to a scarcity in the production of other vaccines since production is focused on a few countries?

The pre-pandemic moment was one characterized by serious health incidents from an epidemiological point of view: measles outbreaks were observed in several continents, with major epidemic outbreaks all over the world. In September 2019, the WHO recorded more than 400,000 reported cases of measles, the highest incidence since 2006^9^. The arrival of the new coronavirus drew attention away from the measles outbreaks and has the potential to compromise programs against other pre-existing diseases and epidemics, such as tuberculosis and malaria. The same happened during the Ebola epidemic^10^. The Strategic Advisory Group of Experts on Immunization (SAGE/WHO) advised that all mass vaccination campaigns in the world should be suspended during the pandemic. Additionally, regular vaccinations against diseases such as measles have been suspended in several countries where resources are being devoted to fight covid-19^6^^,^^9^.

The conditions that encourage the proliferation of covid-19 also increase the spread of other infectious diseases, some of which are vaccine-preventable. In other words, factors such as population density, organization of urban space, and poor sanitary conditions^11^ directly influence both immunization actions and measures to contain the pandemic.

### Reflections from Brazil

Brazil is known for its epidemiological and political specificities, which involve different challenges, (re) actions and strategies when facing vaccine-preventable diseases over time. In the past, Brazil hosted the “vaccine revolt” in 1904, an episode that involved political and ideological disputes. This insurgency was a popular response against authoritarian measures of compulsory smallpox vaccination in the city of Rio de Janeiro^12^. The systematic public actions of immunization in Brazil took shape with the smallpox vaccination campaigns, stimulated by the WHO, at the end of the 1960s. In this political and historical period of military dictatorship, economic advances called the “Brazilian Economic Miracle” did not reduce social inequality, and vaccine campaigns coexisted with the increase in infant mortality^13^. However, the vaccination of famous people and actions in public spaces, disseminated by the mass media, constituted the foundation for the progressive construction of a “culture of immunization.” The popularization of vaccination actions increased demand for such vaccines, and the search for new vaccines strengthened the credibility of national immunization actions. Vaccinations were transformed into a social routine without the use of coercive tactics^12^.

Thus, the *Programa Nacional de Imunizações* (PNI – National Immunization Program), created in 1973 and institutionalized in 1975, strengthened the Brazilian State's regulation and coordination of vaccination actions at national level. Since then, the PNI has offered free and universal vaccines and established the National Vaccination Calendar, considered one of the most extensive in the world^14^^,^^15^. The PNI has achieved sustainability over time by investing in vaccine production and by gaining financial autonomy with public funding, which is guaranteed by law^14^^,^^16^. The progressive increase in vaccine uptake rates over four decades has consolidated PNI as a state policy (not a governmental policy) and has promoted health equity. The PNI is also recognized nationally and internationally^15^.

The Brazilian Unified Health System (SUS), instituted in the political period of re-democratization and promulgation of the 1988 Brazilian Constitution, was guided by the principle that health is a universal right and duty of the State^17^. The expansion of Brazil's Family Health Strategy (FHS) improved the interdependence between primary care units and its immunization program. The FHS was instituted in 1994 as a health care model that guides Brazilian Primary Health Care (PHC), based on multidisciplinary teams, with several health professions. FHS is in accordance with the principles of SUS (universalization, equity and integrality)^18^. Since the emergence of SUS (1988) and the establishment of the FHS, the PNI has been gradually decentralized. It is now present in all the 5,570 Brazilian cities, even the most remote ones^17^.

Despite advances and international recognition, PNI has faced challenges, such as the recent drop in vaccine uptake rates and their considerable differences between regions^19^. The decrease in vaccine uptake rates in Brazil is multifactorial. Some of those factors include the complexity of the PNI; occasional shortages of some vaccines; schedule restrictions of vaccine rooms; the under-financing of SUS; and the dismantling of primary health care^16^^,^^17^. Broader social dimensions also come into play and are found in other countries, such as the increase in vaccine hesitancy and the public's distrust in vaccines, as well as dissemination of content that discourages vaccination, including fake news about the subject^20^. The immediate consequence of the drop in vaccine uptake rates is the return of measles in 2018, which had been eliminated from the country two years prior^21^.

Currently, measures against the spread of covid-19 consist mainly of hygienic actions and social distancing^22^. Recommendations for people to stay home, added to the diversion of human and material resources to fight covid-19, can negatively influence preventive or routine health actions. In the United States of America, vaccination data show a decline in measles-containing vaccines beginning one week after the national covid-19 emergency declaration^23^. In Pakistan, there was a huge decline in the daily average total number of vaccinations administered during lockdown^24^.

Little is known so far about the impact of the covid-19 pandemic on immunization programs and vaccine uptake rates. In May 2020, several Brazilian states enacted restrictions due to the pandemic, from trade closure to lockdown. In the same month, data compiled until May 23 indicated that the country registered 3,629 confirmed cases and four deaths from measles^25^. According to the WHO, by September 2020, Brazil occupied the first place at the Top 10 list of Measles Incidence Rate per Million. The country registered 24,956 cases of measles and an incidence rate of 117.5 from August 2019 to July 2020^26^.

There was a reduction in the demand for vaccine rooms during the pandemic in Brazil, even though the service continues to be offered universally and free of charge within the PHC system^27^. In contrast, some publications report experiences of the Brazilian PHC successfully meeting essential demands, such as immunizations, even with the PHC units being the gateway to suspected cases of covid-19^1^^,^^5^. The PHC Units, which are decentralized and based on geographical territories, intend to get closer to the community, reducing the distance to the health service. Based on an enrolled population, the organization allowed family health teams to assist their patients using other tools, such as telephone and video calls^28^. Additionally, the entry of the units was readjusted to separate suspected cases of covid-19 from patients with different demands, such as vaccination^5^.

The lack of national coordination by the government and the bad example set by the Brazilian President Jair Bolsonaro in responding to covid-19^29^^,^^30^ reveal the conflictual positions between the federal government and governors from the 27 states of the country. Scientific denial results in minimizing the health dangers of covid-19 and discourages social distancing measures^31^. Additionally, the investment in drugs without evidence of effectiveness for the treatment of covid-19 and the concealment of epidemiological information about the pandemic only accentuate the country's weakness in controlling the coronavirus.

These aspects also contribute to the public discrediting and forgoing health actions and inaction in accessing health services in general. The more ineffective the treatments and the longer the fight against SARS-CoV-2 in Brazil, the higher the impact on routine vaccination and vaccine uptake rates^32^. A decrease in confidence in public health actions may affect the country's historic immunization culture, as well as the prestige and credibility of the PNI, which was successfully achieved in its 47 years of existence.

Vulnerable populations are at higher risk of dying, whether is it due to a global pandemic, outbreaks of communicable diseases previously eradicated, or the absence of health and social protection policies^33^. Despite this, Mukwege^34^ believes the time is ripe for social mobilization around public health; for challenging state apparatus and technocratic structures: and for mobilizing local communities and civil societies. The covid-19 pandemic poses and will continue to pose great challenges to health systems and immunization programs around the globe and in Brazil.
